# Intracerebroventricular injection of human umbilical cord blood mesenchymal stem cells in patients with Alzheimer’s disease dementia: a phase I clinical trial

**DOI:** 10.1186/s13195-021-00897-2

**Published:** 2021-09-14

**Authors:** Hee Jin Kim, Kyung Rae Cho, Hyemin Jang, Na Kyung Lee, Young Hee Jung, Jun Pyo Kim, Jung Il Lee, Jong Wook Chang, Seongbeom Park, Sung Tae Kim, Seung Whan Moon, Sang Won Seo, Soo Jin Choi, Duk L. Na

**Affiliations:** 1 Departments of Neurology, Samsung Medical Center, Sungkyunkwan University School of Medicine, 80 Ilwon-ro, Gangnam-gu, Seoul, 135-710 Republic of Korea; 2 Alzheimer’s Disease Convergence Research Center, Samsung Medical Center, Seoul, Republic of Korea; 3 Neuroscience Center, Samsung Medical Center, Seoul, Republic of Korea; 4 Stem Cell & Regenerative Medicine Institute, Samsung Medical Center, Seoul, Republic of Korea; 5 Department of Health Sciences and Technology, SAIHST, Sungkyunkwan University, Seoul, Republic of Korea; 6 Department of Neurosurgery, Konkuk University School of Medicine, Seoul, Republic of Korea; 7 Sungkyunkwan University School of Medicine, Seoul, Republic of Korea; 8 Department of Neurology, Myongji Hospital, Hanyang University, Goyang, Republic of Korea; 9 Center for Neuroimaging, Radiology and Imaging Sciences, Indiana University School of Medicine, Indianapolis, IN USA; 10 Department of Neurosurgery, Samsung Medical Center, Sungkyunkwan University School of Medicine, Seoul, Republic of Korea; 11 Department of Radiology, Samsung Medical Center, Sungkyunkwan University School of Medicine, Seoul, Republic of Korea; 12 Department of Nuclear Medicine, Samsung Medical Center, Sungkyunkwan University School of Medicine, Seoul, Republic of Korea; 13 Biomedical Research Institute, R&D Center, MEDIPOST Co., Ltd., Seongnam, Republic of Korea

**Keywords:** Alzheimer’s disease, Mesenchymal stem cell, Intracerebroventricular injection, Phase I/IIa

## Abstract

**Backgrounds:**

Alzheimer’s disease is the most common cause of dementia, and currently, there is no disease-modifying treatment. Favorable functional outcomes and reduction of amyloid levels were observed following transplantation of mesenchymal stem cells (MSCs) in animal studies.

**Objectives:**

We conducted a phase I clinical trial in nine patients with mild-to-moderate Alzheimer’s disease dementia to evaluate the safety and dose-limiting toxicity of three repeated intracerebroventricular injections of human umbilical cord blood–derived MSCs (hUCB-MSCs).

**Methods:**

We recruited nine mild-to-moderate Alzheimer’s disease dementia patients from Samsung Medical Center, Seoul, Republic of Korea. Four weeks prior to MSC administration, the Ommaya reservoir was implanted into the right lateral ventricle of the patients. Three patients received a low dose (1.0 × 10^7^ cells/2 mL), and six patients received a high dose (3.0 × 10^7^ cells/2 mL) of hUCB-MSCs. Three repeated injections of MSCs were performed (4-week intervals) in all nine patients. These patients were followed up to 12 weeks after the first hUCB-MSC injection and an additional 36 months in the extended observation study.

**Results:**

After hUCB-MSC injection, the most common adverse event was fever (*n* = 9) followed by headache (*n* = 7), nausea (*n* = 5), and vomiting (*n* = 4), which all subsided within 36 h. There were three serious adverse events in two participants that were considered to have arisen from the investigational product. Fever in a low dose participant and nausea with vomiting in another low dose participant each required extended hospitalization by a day. There were no dose-limiting toxicities. Five participants completed the 36-month extended observation study, and no further serious adverse events were observed.

**Conclusions:**

Three repeated administrations of hUCB-MSCs into the lateral ventricle via an Ommaya reservoir were feasible, relatively and sufficiently safe, and well-tolerated. Currently, we are undergoing an extended follow-up study for those who participated in a phase IIa trial where upon completion, we hope to gain a deeper understanding of the clinical efficacy of MSC AD therapy.

**Trial registration:**

ClinicalTrials.gov NCT02054208. Registered on 4 February 2014. ClinicalTrials.gov NCT03172117. Registered on 1 June 2017

**Supplementary Information:**

The online version contains supplementary material available at 10.1186/s13195-021-00897-2.

## Introduction

Alzheimer’s disease (AD), an age-related neurodegenerative disorder, is the most common cause of dementia. Currently, disease-modifying therapies that can change the course of the disease are not yet available. Numerous clinical trials that targeted single patho-mechanisms such as amyloid-beta (Aβ) or tau, which are core pathological features of AD, fell short of robust data to demonstrate the clinical efficacy of single-target therapy. Since AD has multiple patho-mechanisms, multi- instead of single-target therapies may be more effective in shifting the clinical course of the disease.

Mesenchymal stem cells (MSCs) are a promising candidate to treat neurodegenerative disorders including AD [1–5]. When MSCs are injected into the brains of AD mouse models, rather than differentiating into neurons and glial cells, MSCs secrete various cytotrophic factors that can tackle the multiple patho-mechanisms of AD [6]. More specifically, previous preclinical studies showed that soluble intracellular adhesion molecule-1 (sICAM-1) and AgRP secreted by human umbilical cord blood–derived MSCs (hUCB-MSCs) reduced Aβ level [7–9], hUCB-MSC–derived galectin-3 protected against Aβ neurotoxicity [10], and hUCB-MSCs enhanced endogenous adult neurogenesis and synaptic activity via secretion of GDF-15 and Activin A [11]. Based upon these animal studies, we previously completed a phase I clinical trial where we conducted stereotactic injections of hUCB-MSCs into the hippocampus and precuneus of nine AD dementia patients [12]. Through this trial, we first confirmed the safety of MSC therapy. Considering the progressive nature of AD; however, we considered that it would be both crucial and imperative to perform repeated deliveries of MSCs for future studies so as to preserve the therapeutic efficacy of hUCB-MSCs for a prolonged period of time. According to reports that we recently published [13, 14], there was a low persistence of naive human MSCs administered into the parenchyma or lateral ventricle of wild-type mice at 1 week post-transplant. In a mouse model, only 5.7% of human Wharton’s jelly–derived MSCs injected into the intracerebroventricular space persisted a week following transplantation [14]. Many groups have also noted the poor survival of MSCs following administration as a limitation of MSC-based therapy [15, 16]. Such results further reinforced the need to perform repeated injections in order to substitute for the lost MSCs destined for short survival, as reported in a mouse model study [17]. Although we previously reported the safety of parenchymal injection of MSCs, it is too invasive to perform multiple parenchymal injections by applying the stereotactic approach. Thus, to account for the importance of performing repeated injections, an Ommaya reservoir was utilized to deliver the MSCs. The Ommaya reservoir was implanted into the lateral ventricle. Among the various administration routes to deliver MSCs to the brain, the intracerebroventricular route may be advantageous in that a wide distribution of MSCs in the parenchyma can be achieved [18, 19]. It has been reported that MSCs can migrate into the brain parenchyma by initially adhering to the walls of the lateral ventricle [17, 20]. Indeed, we previously demonstrated that hUCB-MSCs transplanted into the intracerebroventricular space of a canine model migrated from the lateral ventricle towards the cortex [21].

Here, we conducted a phase I clinical trial where we evaluated the safety and dose-limiting toxicity of repeated intracerebroventricular injections of hUCB-MSCs in nine patients with mild-to-moderate AD dementia.

## Methods

### Study design

**Fig. 1:**
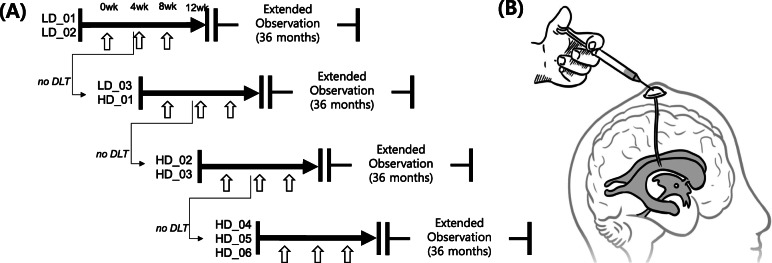
Study flow diagram for a phase I protocol in participants with Alzheimer’s disease. **A** Participants underwent three repeated hUCB-MSC injections in a dose-escalating, open-label fashion with two sequential doses (1.0 × 10^7^ cells/2 mL in the low dose [LD] group and 3.0 × 10^7^ cells/2 mL in the high dose [HD] group). Before continuing the study with subsequent participants, we confirmed that there was no DLT. Empty arrows indicate hUCB-MSC injection. **B** Schematic drawing of right intracerebroventricular injection of hUCB-MSCs via the Ommaya reservoir

This was an open-label, single-center, phase I clinical trial performed at Samsung Medical Center, Seoul, Republic of Korea. First, participants underwent Ommaya reservoir implantation into the right lateral ventricle under local anesthesia, and then 4 weeks later, the participants received their first injection of MSCs followed by an additional two repeated hUCB-MSC injections at 4-week intervals (Fig. 1A, B). The participants received sequential hUCB-MSC (NEUROSTEM®) injections in a dose-escalating manner (Fig. 1). The rationale for the dose of injected hUCB-MSCs is provided in supplementary data. The first two participants (LD_01 and LD_02) received injections of a low dose (1.0 × 10^7^ cells/2 mL) of hUCB-MSCs into the right lateral ventricular space via an Ommaya reservoir. Four weeks after the first injection, the dose-limiting toxicity (DLT) was evaluated. DLT was assessed according to the National Cancer Institute - Common Toxicity Criteria for Adverse Event (NCI-CTCAE) classification which encompassed grade 3 or higher toxicities [22]. After confirming the absence of DLT, the first two participants (LD_01 and LD_02) received their second hUCB-MSC injections, and the next two participants (LD_03 and HD_01) were subsequently enrolled to receive injections of a low (1.0 × 10^7^ cells/2 mL) and high (3.0 × 10^7^ cells/2 mL) dose of MSCs, respectively. During the entire study period, DLT was evaluated 4 weeks after each injection for each participant. We decided to continue repeated injections or enroll the next participants only when the absence of DLT was confirmed.

This trial was registered at ClinicalTrial.gov for 12 weeks of follow-up (Identifier: NCT02054208) and for 36 months of extended observation (Identifier: NCT03172117). We obtained written informed consent from every patient and legally authorized representatives in cases of impaired capacity. This study was approved by the Institutional Review Board of Samsung Medical Center and Ministry of Food and Drug Safety (**MFDS)**.

### Participants

We enrolled nine AD dementia patients at Samsung Medical Center from March 2014 to June 2017. Eligible patients were 50 to 85 years of age, fulfilled the criteria for probable AD dementia according to the National Institute on Aging-Alzheimer’s Association workgroup (NIA-AA) [23], and had a Mini-Mental State Examination (MMSE) score of 18–26 at screening. Eligible patients were further screened with amyloid positron emission tomography (PET), [^18^F] fluoro-2-deoxy-d-glucose (FDG) PET, and magnetic resonance imaging (MRI) and were enrolled when they satisfied the following criteria: (1) had significant amyloid burden assessed by ^11^C-Pittsburgh compound B (PiB) PET or ^18^F-Florbetaben PET and (2) had neuronal degeneration assessed by atrophy on structural brain MRI or hypometabolism on FDG PET.

Patients with one or more of the following conditions were excluded: (1) neurodegenerative diseases other than AD dementia; (2) severe white matter hyperintensities on fluid-attenuated inversion recovery (FLAIR) MR images at baseline, which were defined as a cap or band (periventricular white matter hyperintensities) ≥ 10 mm, and deep white matter lesions (deep white matter hyperintensities) ≥ 25 mm as modified from the Fazekas ischemia criteria [24]; (3) major psychiatric disorders; (4) history of stroke within 3 months of enrollment; (4) hepatic, renal, hematologic, or active pulmonary disorders; (5) current or previous history of malignancy; and (6) difficult to perform Ommaya reservoir insertion. Patients were required to have sustained a stable dose of acetylcholinesterase inhibitors or memantine for at least 60 days prior to enrollment and the same dose of medication was continued throughout the study.

### Preparation of hUCB-MSCs

To be used for clinical purposes, hUCB-MSC manufacture, cell quality control, and quality assurance were performed in compliance with the Korea Good Manufacturing Practices requirements by the MFDS. hUCB tissue was obtained after receiving written informed consent from normal women in their full-term pregnancy. hUCB-MSCs were grown in α-Minimum Essential Medium (α-MEM, Gibco/Life Technologies, Carlsbad, CA, USA) supplemented with 10% fetal bovine serum (Gibco/Life Technologies). Cells were cryopreserved at − 150 °C or lower by using 10% dimethyl sulfoxide.

To prepare for intracerebroventricular injection, frozen hUCB-MSCs were first thawed, seeded, and cultured. The cells were harvested 5 days after seeding, repeatedly washed to remove impurities such as fetal bovine serum and trypsin, and re-suspended in an appropriate amount of phenol red-free α-MEM. Therefore, fetal bovine serum was not present in the final drug product, NEUROSTEM®. hUCB-MSCs were tested for viability, phenotype, and presence of endotoxins, bacteria, and mycoplasma. After testing, 50 million cells were re-suspended in 1 mL of phenol red-free α-MEM, and a total volume of 2 mL was used to inject MSCs into a single patient of the low dose group. For the high dose group, from the adjusted final concentration of 150 million cells per 1 mL of phenol red-free MEM-α, a total volume of 2 mL was prepared for administration into each individual patient. hUCB-MSCs for the low dose group were stored at 4–20 °C and had a shelf life of about 60 h from the time of manufacture, and hUCB-MSCs for the high dose group were stored at 4–12 °C and had a shelf life of about 48 h. Based on flow cytometric analyses, the expressions of surface antigens of the cells were consistently positive for CD73, CD90, CD105, and CD166 but negative for CD45, CD14, and HLA-DR. The final products were delivered to the patient’s physician on the day of injection.

### Ommaya insertion and intracerebroventricular injection of hUCB-MSCs

Thin-sliced brain CT scans of each patient were acquired for intraoperative navigation before surgery. Under local anesthesia, burr holes (approximately 15 mm) were made in the patient’s right Kocher’s point. An electromagnetic navigation system was used to guide the pathway to the lateral ventricle to avoid possible misplacement of the catheter and intra-parenchymal hemorrhage. The catheter was inserted about 6 cm from the dura opening and was connected to the reservoir chamber (Integra, NJ, USA). To assure that the catheter was securely inserted inside the lateral ventricle, cerebrospinal fluid (CSF) filling following pumping of the reservoir was checked, and intraoperative CT scans were also taken.

The average time interval between screening and Ommaya insertion was 13.7.0 ± 7.3 days. Four weeks following Ommaya reservoir implantation, the participants were admitted to the hospital and received baseline evaluation in the morning, and in the afternoon, a neurologist injected hUCB-MSCs into the intracerebroventricular space via the Ommaya reservoir (low dose 1.0 × 107 cells/2 mL or high dose 3.0 × 107 cells/2 mL). The participants were hospitalized for 2 days and were observed for signs of acute adverse events.

### Safety assessments

Our primary goal was to assess the safety and DLT of two ascending doses of hUCB-MSCs in a total of three repeated injections. Safety was evaluated at each visit by examining the vital signs and weight, physical and neurologic examination, electrocardiogram, and clinical laboratory testing (hematologic and serum chemical testing). DLT was evaluated 4 weeks after each hUCB-MSC injection. Adverse events were assessed at each visit according to NCI-CTCAE [22]. In the extended observation study, adverse events were assessed at 18, 24, and 36 months after the end of the phase I trial. C&R Research Inc. served as external monitors of the study.

To detect immunologic reactions between the implanted hUCB-MSCs and the recipient, a mixed lymphocyte reaction was assessed at baseline and at 12 weeks after the first hUCB-MSC injection.

In order to monitor the complications (e.g., parenchymal or intraventricular hemorrhage) related to the Ommaya reservoir implantation, patients underwent brain CT scans at 24 h and 4 weeks after insertion of the Ommaya reservoir.

### Clinical and laboratory assessments

To measure the clinical changes, patients underwent 11-item AD Assessment Scale-Cognitive Subscale (ADAS-Cog 11) testing (score range 0–70, lower score indicates better performance) [25], Seoul Instrumental Activities of Daily Living (S-IADL) (score range 0–45, lower score indicates better performance) [26], MMSE (score range 0–30, higher score indicates better performance), Clinician’s Interview-Based Impression of Change Plus Caregiver Input (CIBIC-Plus) [27], and Caregiver-Administered Neuropsychiatric Inventory (CGA-NPI) (sum of frequency × severity of 12 behavioral domains, score range 0–144, higher score indicates worse behavior) [28] at baseline, 4 weeks, 8 weeks, and 12 weeks after the first hUCB-MSC injection. In the extended observation study, the same assessment was done at 12 and 24 months after the end of the phase I trial.

We evaluated the changes in CSF white blood cell (WBC) count, AD biomarkers (CSF Aβ, total-tau, and phosphorylated-tau), and hUCB-MSC–related markers (Galactine-3, sICAM-1, progranulin, GDF-15, and Decorin) at baseline, 1 day, and 4 weeks after the 1st hUCB-MSC injection, 1 day and 4 weeks after the 2nd hUCB-MSC injection, and 1 day and 4 weeks after the 3rd hUCB-MSC injection. CSF was collected via the Ommaya reservoir.

To evaluate the structural changes, patients underwent brain MRI at baseline and 12 weeks after the first hUCB-MSC injection and at 12 and 24 months after the end of the phase I trial. To measure the changes in parenchymal Aβ deposition, patients underwent PiB-PET or florbetaben PET at baseline and 12 weeks after the first hUCB-MSC injection. Changes in standardized uptake value ratio (SUVR) were assessed.

### Statistical analysis

Due to the small number of participants, absence of sham-surgery-control, and the fact that it was an open-labeled study design, we did not perform statistical analysis to compare the outcome measures of both the low- and high-dose hUCB-MSC recipients.

## Results

### Compliance and safety

The baseline characteristics of each individual subject are listed in Table 1. The mean age was 62.3 years in the low dose group and 63.0 years in the high dose group. All of the enrolled patients completed the phase I 12-week follow-up trial. For the extended follow-up study, three patients (LD_01, LD_03, and HD_04) declined to participate, and thus, only six out of nine patients were enrolled. During the extended follow-up study, one patient (LD_02) also dropped out of the study due to withdrawal of consent, and another patient (HD_06) was not included in the final clinical assessment at 24 months because the patient refused.

**Table 1:** Baseline characteristics of participants

Subject	Age	Gender	Education, years	Medication	MMSE	ADAS-Cog	APOE genotype	Amyloid PET
LD_01	59	M	9	Donepezil 5 mg	21	19	e3/e4	Positive
LD_02	65	F	12	Donepezil 10 mg	26	19	e3/e3	Positive
LD_03	63	F	12	Donepezil 10 mg	25	16	e4/e4	Positive
HD_01	60	M	18	Donepezil 10 mg	18	20	e3/e4	Positive
HD_02	76	F	12	Donepezil 7.5 mg	17	27	e3/e3	Positive
HD_03	50	F	14	Rivastigmine 9.5 mg/24 h	14	29	e3/e3	Positive
HD_04	66	F	6	Memantine 10 mg, donepezil 10 mg	24	19	e3/e4	Positive
HD_05	53	F	16	Donepezil 10 mg	24	13	e3/e3	Positive
HD_06	73	M	12	Rivastigmine 9.5 mg/24 h	21	29	e4/e4	Positive

*LD*, low dose; *HD*, high dose; *AChE-I*, acetylcholine esterase inhibitor; *MMSE*, Mini-Mental State Examination; *ADAS-Cog*, Alzheimer’s Disease Assessment Scale-Cognitive Subscale

The Ommaya reservoir insertion procedure was well tolerated in all nine participants. However, Ommaya site pain or procedural pain occurred in four participants, and headache occurred in one participant. Brain CT scans taken within 24 h after Ommaya reservoir insertion confirmed that there was no cerebral hemorrhage in any of the participants.

After each hUCB-MSC injection, the participants were hospitalized for 2 days to be monitored for acute adverse events. After hUCB-MSC injection, all nine (100%) participants experienced fever. Out of a total of nine low-dose injections (three repeated injections for three participants), fever occurred after seven injections. Out of a total of 18 high-dose injections (three repeated injections for six participants), fever occurred after 13 injections. Fever subsided within 1–2 days with oral or IV antipyretics, and no antibiotics or antiviral agents were necessary. Other acute adverse events were headaches 7/9 (77.8%), followed by nausea 5/9 (55.6%), vomiting 4/9 (44.4%), myalgia or chills 2/9 (22.2%), and paresthesia on both arms and legs 2/9 (22.2%). Other events were abdominal pain 1/9 (11.1%), dyspepsia 1/9 (11.1%), pain other than the Ommaya site 1/9 (11.1%), musculoskeletal stiffness 1/9 (11.1%), arthralgia 1/9 (11.1%), periarthritis 1/9 (11.1%), and impetigo 1/9 (11.1%) (Table 2).

**Table 2:** Adverse events that were observed in at least 1 subject during the 12-week follow-up period (all causalities)

	Low dose (***n*** = 3)	High dose (***n*** = 6)
	No. of subjects (%) [no. of events]	No. of subjects (%) [no. of events]
**Before hUCB-MSC injection**		
Total	2 (66.7) [4]	5 (83.3) [5]
Ommaya site pain	0 [0]	2 (33.3) [2]
Procedural pain	1 (33.3) [1]	1 (16.7) [1]
Headache	1 (33.3) [2]	0 [0]
Device malfunction	0 [0]	1 (16.7) [1]
Enteritis	1 (33.3) [1]	0 [0]
Otolithiasis	0 [0]	1 (16.7) [1]
**After hUCB-MSC injection**		
Total	3 (100.0) [21]	6 (100.0) [48]
Nervous system disorders		
Headache	2 (66.7) [5]	5 (83.3) [12]
Paraesthesia	0 [0]	2 (33.3) [4]
Gastrointestinal disorders		
Nausea	2 (66.7) [E2]	3 (50.0) [7]
Vomiting	1 (33.3) [2]	3 (50.0) [6]
Abdominal pain	0 [0]E	1 (16.7) [1]
Dyspepsia	1 (33.3) [1]	0 [0]
General disorders and administration site conditions		
Fever	3 (100) [7]	6 (100) [13]
Chills	0 [0]	1 (16.7) [1]
Ommaya site pain	1 (33.3) [1]	0 [0]
Pain other than the Ommaya site	0 [0]	1 (16.7) [1]
Musculoskeletal and connective tissue disorders		
Musculoskeletal stiffness	0 [0]	1 (16.7) [1]
Myalgia	1 (33.3) [2]	0 [0]
Arthralgia	0 [0]	1 (16.7) [1]
Periarthritis	0 [0]	1 (16.7) [1]
Infections and infestations		
Impetigo	1 (33.3) [1]	0 [0]
Psychiatric disorders		
Delusion	0 [0]	0 [0]

Among the aforementioned adverse events, three serious adverse drug reactions occurred in two participants. A 59-year-old male in the low dose group (LD_01) experienced a 38.0 °C fever 1 day after the second hUCB-MSC injection, which extended hospitalization by a day for close observation. One day after the third hUCB-MSC injection, the patient experienced a 38.6 °C high fever, which again extended hospitalization by a day. In both events, the fever subsided the next day without the use of antibiotics or anti-viral medication. According to CTCAE, the degree of adverse event was grade 1 and deemed by investigators as probably related to the investigational product. The second patient was a 65-year-old female who experienced nausea and vomiting 1 day after the second low-dose (LD_02) hUCB-MSC injection, which extended hospitalization by a day for close observation. The symptom subsided the next day without specific treatment. According to CTCAE, the degree of adverse event was grade 1 and deemed by investigators as probably related to the investigational product.

Brain MR images that were taken 12 weeks after the first hUCB-MSC injection, and at 12 and 24 months during extended observation, confirmed that there were no structural abnormalities including tumor, hydrocephalus, or subdural hemorrhages. Furthermore, dose-limiting toxicities were not evident for either the low- or high-dose hUCB-MSC groups. The results of the mixed lymphocyte reaction performed at 12 weeks after the first hUCB-MSC injection showed that all nine subjects were immunologically stable.

### Clinical and laboratory outcome measures

**Fig. 2:**
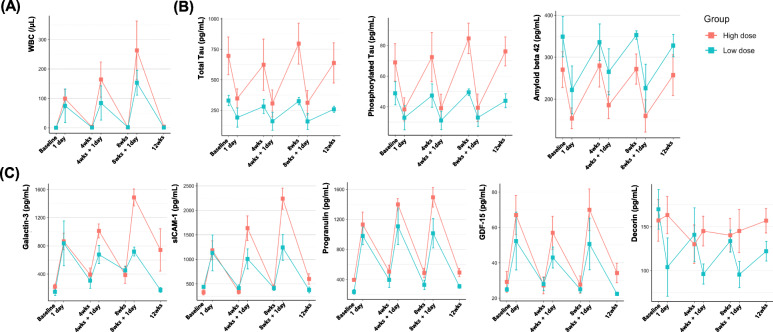
White blood cell count (**A**), Alzheimer’s disease biomarkers (**B**), and MSC-related markers (**C**) in the cerebrospinal fluid. Bars indicate standard error. WBC, white blood cell; sICAM-1, soluble intercellular adhesion molecule-1; GDS-15, growth differentiation factor 15

Scores of ADAS-Cog, MMSE, SIADL, and NPI at each time point are shown in Table 3. The number of patients in each category of CIBIC-Plus is shown in Table 4. CSF WBC count increased 1 day after each hUCB-MSC injection which then normalized after 4 weeks. The WBC increment amplified on repeated injections and a higher WBC increment were observed from the high dose group (Fig. 2A).

**Table 3:** Scores of ADAS-Cog, S-IADL, MMSE, and NPI at each time point

`		ADAS-Cog			S-IADL			MMSE			NPI		
		Low dose	High dose	Total	Low dose	High dose	Total	Low dose	High dose	Total	Low dose	High dose	Total
		*n* = 3	*n* = 6	*n* = 9	*n* = 3	*n* = 6	*n* = 9	*n* = 3	*n* = 6	*n* = 9	*n* = 3	*n* = 6	*n* = 9
Screening	Mean ± SD	15.0 ± 2.0	22.5 ± 6.2	20.0 ± 6.3	9.3 ± 4.0	13.0 ± 5.8	11.8 ± 5.3	24.0 ± 1.7	21.3 ± 2.7	22.2 ± 2.6	10.0 ± 7.8	1.5 ± 1.5	4.3 ± 5.9
	Median	15	23	19.7	10	14.7	13	24	20.7	22	6	1.3	2.7
	Min–Max	13–17	12–30	12–30	5–13	5–20	5–20	22–25	18–25	18–25	5–19	0–4	0–19
Baseline	Mean ± SD	18.0 ± 1.7	22.8 ± 6.5	21.2 ± 5.8	8.3 ± 4.6	15.3 ± 5.9	13.0 ± 6.3	24.0 ± 2.6	19.7 ± 4.0	21.1 ± 4.1	1.0 ± 1.7	5.3 ± 8.9	3.9 ± 7.4
	Median	18	23.5	19.5	8.3	17.5	13	25	19.5	21.8	1	1.5	1
	Min–Max	16–19	13–29	13–29	3–11	7–21	3–21	21–26	14–24	14–26	0–3	0–23	0–23
Week 4	Mean ± SD	17.3 ± 3.8	22.2 ± 5.5	20.6 ± 5.3	12.3 ± 5.9	16.5 ± 9.4	15.1 ± 8.2	24.3 ± 1.5	21.5 ± 3.3	22.4 ± 3.1	5.7 ± 2.3	3.5 ± 3.6	4.2 ± 3.2
	Median	19	21.3	19.3	10	15.5	12	24	21.5	23.3	5.7	3	4
	Min–Max	13–20	16–29	13–29	8–19	5–28	5–28	23–26	17–25	17–26	3–7	0–9	0–9
Week 8	Mean ± SD	15.7 ± 6.1	23.2 ± 6.7	20.7 ± 7.2	10.3 ± 2.1	17.8 ± 6.3	15.3 ± 6.3	26.0 ± 2.0	21.5 ± 3.6	23.0 ± 3.7	5.0 ± 5.6	5.3 ± 6.7	5.2 ± 6.0
	Median	17	24	20.3	11	17.5	13	26	22	23.5	4	4	4
	Min–Max	9–21	13–31	9–31	8–12	10–28	8–28	24–28	16–26	16–28	0–11	0–18	0–18
Week 12	Mean ± SD	18.7 ± 2.5	25.2 ± 8.2	23.0 ± 7.4	10.3 ± 5.0	16.8 ± 6.5	14.7 ± 6.6	24.0 ± 4.4	20.3 ± 3.6	21.6 ± 4.0	2.3 ± 2.1	3.7 ± 6.0	3.2 ± 4.9
	Median	19	23.5	21	11	17	14	26	20	21	3	3.2	2
	Min–Max	16–21	17–36	16–36	5–15	8–26	5–26	19–27	16–25	16–27	0–4	0–14	0–14
Mean change from baseline to week 12	Mean ± SD	0.7 ± 4.0	2.3 ± 5.0	1.8 ± 4.5	2.0 ± 2.0	1.5 ± 3.0	1.7 ± 2.6	0.0 ± 2.0	0.7 ± 1.6	0.4 ± 1.7	1.3 ± 2.3	− 1.7 ± 5.8	− 0.7 ± 5.0
	Median	0	3.5	3	2	1.5	1.7	0	0.5	0.3	1.3	− 1.5	− 0.5
	Min–Max	− 3–5	− 4–9	− 4–9	0–4	− 3–6	− 3–6	− 2–2	− 1–3	− 2–3	0–4	− 9–8	− 9–8
Week 52		*n* = 1	*n* = 5	*n* = 6	*n* = 1	*n* = 5	*n* = 6	*n* = 1	*n* = 5	*n* = 6	*n* = 1	*n* = 5	*n* = 6
	Mean ± SD	19	30.0 ± 9.8	28.2 ± 9.8	17	23.4 ± 9.0	22.3 ± 8.4	23	16.0 ± 6.0	17.2 ± 6.0	41	2.6 ± 2.1	9.0 ± 15.8
	Median	19	31.0	28.5	17	21	19.5	23	17.0	18.5	41	3.0	3.5
	Min–Max		16–42	16–42		14–37	14–37		6–21	6–23		0–5	0–41
Week 104		*n* = 0	*n* = 4	*n* = 4	*n* = 0	*n* = 4	*n* = 4	*n* = 0	*n* = 4	*n* = 4	*n* = 0	*n* = 4	*n* = 4
	Mean ± SD		35.3 ± 13.6	35.3 ± 13.6		30.0 ± 9.2	30.0 ± 9.2		13.3 ± 8.0	13.3 ± 8.0		0.8 ± 1.0	0.8 ± 1.0
	Median		32	32		31	31		14	14		0.7	0.7
	Min–Max		23–54	23–54		18–40	18–40		3–22	3–22		0–2	0–2

*ADAS-Cog*, Alzheimer’s Disease Assessment Scale-Cognitive Subscale; *S-IADL*, Seoul Instrumental Activities of Daily Living; *MMSE*, Mini-Mental State Examination; *NPI*, neuropsychiatric inventory; *hUCB-MSC*, human umbilical cord blood–derived mesenchymal stem cell; *SD*, standard deviation

**Table 4:** Number of patients in each category of CIBIC-Plus

	Baseline		Week 4		Week 8		Week 12	
	Low dose	High dose	Low dose	High dose	Low dose	High dose	Low dose	High dose
Marked worsening								
Moderate worsening				1		1		3
Minimal worsening		2	2			1		
No change	3	3		2		2	2	1
Minimal improvement		1	1	1	2	2	1	1
Moderate improvement				1	1			1
Marked improvement				1				

*CIBIC-Plus*, Clinician’s Interview-Based Impression of Change Plus Caregiver Input

AD biomarkers (total tau, phosphorylated tau, and Aβ42) decreased 1 day after each hUCB-MSC injection which then increased to the baseline level after 4 weeks (Fig. 2B).

hUCB-MSC–related markers (Galactin-3, sICAM-1, progranulin, and GDF-15) increased 1 day after each hUCB-MSC injection which then decreased to the baseline level after 4 weeks. However, such patterns were not observed from the CSF decorin levels (Fig. 2C).

Aβ burden measured by amyloid-PET showed that in two participants of the low dose group, PiB SUVR changed from 2.57 ± 0.40 at baseline to 1.92 ± 0.03 at 12-week follow-up; in one participant of the low dose group, florbetaben SUVR changed from 1.70 at baseline to 1.60 at 12-week follow-up; and in six participants in the high dose group, florbetaben SUVR changed from 1.79 ± 0.07 at baseline to 1.71 ± 0.06 at 12-week follow-up.

## Discussion

In this phase I clinical trial, we repeatedly injected hUCB-MSCs into the right lateral ventricle of nine mild-to-moderate AD dementia patients via an Ommaya reservoir. Three patients received a low dose (1.0 × 10^7^ cells/2 mL) and six patients received a high dose (3.0 × 10^7^ cells/2 mL) of hUCB-MSCs 3 times at 4-week intervals. We report that this was feasible, safe, and well-tolerated. However, within 24 h after each MSC administration, we consistently observed transient fever and increased WBC count in the CSF samples of the patients. The fever subsided after 1–2 days, and WBC was normalized by the next CSF examination that took place 4 weeks later.

First, in regard to safety, side effects associated with the Ommaya reservoir implantation were not remarkable. Implantation was performed under local anesthesia and was well tolerated in all patients. Following surgery, minor adverse events such as pain at the site of Ommaya reservoir implantation, procedural pain, and headaches were noted, but major complications such as hemorrhages or infections were not observed. Second, with respect to the complications related to the intracerebroventricular injection of hUCB-MSC into the lateral ventricle, the most common adverse events were fever followed by headache, nausea, vomiting, myalgia, or chills. Three events in two participants were assessed as serious adverse drug reactions as the event extended hospitalization by 1 day. However, the symptom subsided the next day without complications. In the extended observation study, serious adverse drug reactions were not observed. No structural abnormalities such as tumor development, hydrocephalus, or hemorrhage were observed from the five participants who completed the extended observation. These results are consistent with our previous study where we reported the safety of performing stereotactic brain injections of hUCB-MSC in AD patients [12].

Unexpectedly, however, all of the participants developed fever within 6–24 h after receiving hUCB-MSC injections which subsided within the next 36 h. This was surprising because in our previous study, fever was not observed following stereotactic injections of hUCB-MSCs into the hippocampus and precuneus [12]. We presumed that infection, composition of the suspension media, or the MSCs themselves could have caused the fever. However, out of the three possible explanations, infection seemed unlikely since microorganisms were not identified from the CSF culture and the fever subsided spontaneously without the use of antibiotics or antiviral agents. The second hypothesis was that MSCs per se caused the fever. Increased WBC count in the CSF was accompanied by fever which normalized by the next CSF test performed 4 weeks later, right before performing another round of MSC injections. The WBC count was higher for the high dose group. Since we carried out repeated hUCB-MSC injections, the increment of WBC count also amplified. This suggests that immune responses against hUCB-MSCs were sensitized in the second and third injections. We also noted that an increase in the dose of MSC injection led to an increase in WBC count. Although the inflammatory response was transient, we do acknowledge that repeated injections of MSCs could possibly lead towards widespread encephalopathy. Further studies are warranted to find the optimal number of repeated MSC injections considering the repeated inflammatory response and potential therapeutic effects. In addition, we need further studies to search for ways to prolong the survival of MSCs and to thus sustain their therapeutic effects. Mitigating immune responses generated from the transplanted MSCs must also be addressed to enhance the efficacy of stem cell therapy. Evaluating such factors will contribute towards the development of AD stem cell therapy.

This immune reaction was not expected since MSCs are known to express low levels of the major histocompatibility complex (MHC) class I and negative or no levels of MHC class II [13, 29]. However, based on the following observations, we suspect that immune reactions occurred following the administration of hUCB-MSCs. We recently reported that a high infiltration of immune cells was discovered at the injection site a week after performing the administration of human MSCs into the parenchyma or caudate putamen of wild-type mice [13]. While the most prominent and highest expression of immune cells was identified from the group that received transplantation of xenogeneic MSCs, compared to the syngeneic group, mice from the allogeneic group also demonstrated a pronounced expression of immune cells at the injection site [13]. Another group also demonstrated that allo-reactivity was discovered following the administration of bone marrow–derived allogeneic MSCs into the caudate nucleus of immuno-competent rhesus macaques [30]. Interestingly, the extent of host allo-immune response varied depending on the degree of MHC class I and II mismatch [30]. These findings along with the results acquired from this current study challenge the presumption that MSCs are intrinsically immune-privileged. The third possibility was that certain components of the MSC culture media (α-MEM) might have elicited chemical or foreign body reactions. Although further study is required to rule out this possibility, it seems unlikely that α-MEM is the culprit since the amount of α-MEM was identical for both the low and high dose groups. Furthermore, in our mouse experiment, when α-MEM was injected into the parenchyma of wild-type mice, a scarcity of immune cell infiltration was exhibited at the injection site [13].

As this was an open-label phase I clinical trial, we could not prove the clinical efficacy of hUCB-MSC injection. Nevertheless, we noted reduced levels of total tau, phosphorylated tau, and Aβ42 in the CSF samples collected 1 day after performing the hUCB-MSC injections. The increase in total tau, phosphorylated tau, and Aβ42 levels observed 4 weeks following hUCB-MSC transplantation suggest that the therapeutic effects exerted by the hUCB-MSCs may not have lasted for a prolonged period due to the short life span of the hUCB-MSCs. It is interesting to point out that while a striking increase in the expression levels of Galactin-3, sICAM-1, progranulin, and GDF-15 was noted a day following MSC transplantation, the levels dropped dramatically within 4 weeks. Such results support that performing repeated administrations (possibly shortening the interval between the injections) may be necessary to preserve the therapeutic efficacies exerted by the hUCB-MSCs. We also cannot rule out potential dilution effects considering that normal saline was administered right after each injection of hUCB-MSCs. Therefore, CSF biomarker changes observed from the hUCB-MSC injection group should be compared to those of the placebo group in future studies. Placebo-controlled, randomized phase IIa clinical trial of 36 patients is currently under extended follow-up.

### Limitations

A limitation of this study is that the hUCB-MSC injection dose used in this trial was based on mouse studies. The injection dose in this trial was calculated by referring to the mouse to human CSF volume ratio. However, detailed studies to evaluate the optimal cell dose in AD patients are further warranted.

## Conclusions

In this phase I clinical trial, we repeatedly injected hUCB-MSCs into the right lateral ventricle of nine mild-to-moderate AD dementia patients via an Ommaya reservoir. We report that this was feasible, safe, and well-tolerated. However, we consistently observed transient fever following each MSC administration, which subsided after 1–2 days. To date, we are currently conducting an extended follow-up study for those who participated in a placebo-controlled, randomized phase IIa clinical trial. Upon completion, ensuing analyses will be published, and through the analyses, we hope to gain a deeper understanding of the clinical therapeutic efficacy of MSC AD therapy.

## Supplementary Information

**Additional file 1.** The optimal injection dose of hUCB-MSCs.

## Data Availability

The datasets used and/or analyzed during the current study are available from the corresponding author on reasonable request.

## Abbreviations

AD: Alzheimer’s disease
Aβ: Amyloid-beta
MSCs: Mesenchymal stem cells
hUCB-MSCs: Human umbilical cord blood–derived MSCs
DLT: Dose-limiting toxicity
NCI-CTCAE: National Cancer Institute - Common Toxicity Criteria for Adverse Event
MFDS: Ministry of Food and Drug Safety
NIA-AA: National Institute on Aging-Alzheimer’s Association workgroup
MMSE: Mini-Mental State Examination
PET: Positron emission tomography
FDG: Fluoro-2-deoxy-d-glucose
MRI: Magnetic resonance imaging
PIB: ^11^C-Pittsburgh compound B
FLAIR: Fluid-attenuated inversion recovery
α-MEM: α-Minimum Essential Medium
CSF: Cerebrospinal fluid
ADAS-Cog: Alzheimer’s Disease Assessment Scale-Cognitive Subscale
S-IADL: Seoul Instrumental Activities of Daily Living
CIBIC-Plus: Clinician’s Interview-Based Impression of Change Plus Caregiver Input
CGA-NPI: Caregiver-Administered Neuropsychiatric Inventory
WBC: White blood cell
SUVR: Standardized uptake value ratio
MHC: Major histocompatibility complex
