# Supplementary material for: Intracerebroventricular injection of human umbilical cord blood mesenchymal stem cells in patients with Alzheimer’s disease dementia: a phase I clinical trial
Source: Alzheimers Res Ther. 2021 Sep 14;13:154. doi: 10.1186/s13195-021-00897-2 (PMC8439008; doi:10.1186/s13195-021-00897-2)
Supplement: Supplementary file 1 — Additional file 1. The optimal injection dose of hUCB-MSCs. [file 13195_2021_897_MOESM1_ESM.docx]

**Intracerebroventricular Injection of Human Umbilical Cord Blood Mesenchymal Stem Cells in Patients with Alzheimer’s Disease Dementia: A Phase I Clinical Trial**

**The optimal injection dose of hUCB-MSCs**

The rationale of our study was based on animal experiments that we have performed in the past and articles presented by researchers. Toxicity experiments were conducted according to good laboratory practice (GLP) standards and the safety of the injection dosages used in this study was confirmed.

We examined the therapeutic efficacy of hUCB-MSCs in an AD transgenic mouse model (APP/PS1) where we discovered that hUCB-MSC derived GDF-15 promoted endogenous adult neurogenesis [1]. In this particular study we performed repeated injections (a total of 3) of hUCB-MSCs (1.0$\times$10^5^ cells/head/administration) into the cisterna magna of APP/PS1 mice at 4-week intervals. It has been reported that the CSF volume of mice is 35 μL while that of humans is 1000 mL [2]. The CSF volume of humans is approximately 2800 times greater than that of mice. Thus, for a single administration of 1.0$\times$10^5^ cells/head in mice, the equivalent cell concentration will be approximately 280 million cells (2.8$\times$10^8^ cells/administration) in human subjects. This dose is close to 9 times greater than the number of cells injected into the high dose group of our study: 30 million (3.0$\times$10^7^) cells. We also investigated that when 1.0$\times$10^5^ hUCB-MSCs, that were suspended in a larger volume of 7 µL, were injected into the intracerebroventricular space, this enhanced the overall migratory capability and viability of hUCB-MSCs [3]. This is equivalent to the number of hUCB-MSCs injected into the high dose

References

1. Kim DH, Lee D, Chang EH, Kim JH, Hwang JW, Kim JY, et al. GDF-15 secreted from human umbilical cord blood mesenchymal stem cells delivered through the cerebrospinal fluid promotes hippocampal neurogenesis and synaptic activity in an Alzheimer's disease model. Stem Cells Dev. 2015;24:2378-90.

2. Pardridge WM. CSF, blood-brain barrier, and brain drug delivery. Expert Opin Drug Deliv. 2016;13:963-75.

3. Kim HS, Lee NK, Yoo D, Lee J, Choi SJ, Oh W, et al. Lowering the concentration affects the migration and viability of intracerebroventricular-delivered human mesenchymal stem cells. Biochem Biophys Res Commun. 2017;493:751-7.
